# Hypoglycemia Detection Using Hand Tremors: Home Study of Patients With Type 1 Diabetes

**DOI:** 10.2196/40990

**Published:** 2023-04-19

**Authors:** Reza Jahromi, Karim Zahed, Farzan Sasangohar, Madhav Erraguntla, Ranjana Mehta, Khalid Qaraqe

**Affiliations:** 1 Industrial and Systems Engineering Texas A&M University College Station, TX United States; 2 Department of Computer Science and Engineering Texas A&M University College Station, TX United States; 3 Center for Critical Care Houston Methodist Hospital Houston, TX United States; 4 Texas A&M University at Qatar Doha Qatar

**Keywords:** acceleration, hand tremors, tremor, hypoglycemia, blood sugar, glucose, diabetes, diabetic, noninvasive, measurement tool, digital measurement, monitoring, wearable, accelerometer, machine learning, wearable device, smart watch, detect, time domain, frequency domain, model, algorithm

## Abstract

**Background:**

Diabetes affects millions of people worldwide and is steadily increasing. A serious condition associated with diabetes is low glucose levels (hypoglycemia). Monitoring blood glucose is usually performed by invasive methods or intrusive devices, and these devices are currently not available to all patients with diabetes. Hand tremor is a significant symptom of hypoglycemia, as nerves and muscles are powered by blood sugar. However, to our knowledge, no validated tools or algorithms exist to monitor and detect hypoglycemic events via hand tremors.

**Objective:**

In this paper, we propose a noninvasive method to detect hypoglycemic events based on hand tremors using accelerometer data.

**Methods:**

We analyzed triaxial accelerometer data from a smart watch recorded from 33 patients with type 1 diabetes for 1 month. Time and frequency domain features were extracted from acceleration signals to explore different machine learning models to classify and differentiate between hypoglycemic and nonhypoglycemic states.

**Results:**

The mean duration of the hypoglycemic state was 27.31 (SD 5.15) minutes per day for each patient. On average, patients had 1.06 (SD 0.77) hypoglycemic events per day. The ensemble learning model based on random forest, support vector machines, and k-nearest neighbors had the best performance, with a precision of 81.5% and a recall of 78.6%. The results were validated using continuous glucose monitor readings as ground truth.

**Conclusions:**

Our results indicate that the proposed approach can be a potential tool to detect hypoglycemia and can serve as a proactive, nonintrusive alert mechanism for hypoglycemic events.

## Introduction

Diabetes is a chronic condition that is estimated to affect over 9.3% of the global population as of 2019 [1], resulting in the death of 12% of the US population [2] and an estimated US $327 billion in economic costs each year [3]. About 10% of the population with diabetes have type 1 diabetes mellitus (T1DM), and the remaining 90% have type 2 diabetes mellitus [4]. Regular blood sugar monitoring and special attention to food intake are critical to managing diabetes [5,6].

Low blood glucose (BG), also known as hypoglycemia, is a serious condition that affects patients with diabetes when their BG level falls below 70 mg/dL [7]. This is more common for patients with T1DM [8]. Values below 54 mg/dL may cause severe hypoglycemia, leading to cognitive impairment, seizure, and even loss of consciousness [9].

The most prevalent method of monitoring BG has been via BG meters, which require manually pricking the finger to get a reading. However, the main limitation of these meters is that the measurement is periodic and manual. Continuous glucose monitors (CGMs) were commercialized at the beginning of the 21st century [10] and have gained popularity, especially among patients with T1DM [11], as they are capable of monitoring BG levels continuously and autonomously. CGMs can provide information about BG trends and warn against the onset of both hyper- and hypoglycemia. However, despite their benefits, many generations of CGMs have several drawbacks. Although CGMs automatically read BG at short intervals, multiple daily finger sticks are necessary to calibrate the CGM for accuracy [12]. CGMs are usually intrusive, and many patients face barriers to the adoption and continuous use of CGM systems, such as pain, complexity, the need for frequent sensor changes, and frequent calibrations [13]. Newer generations of CGMs (such as Dexcom G6) are less irritating and do not require finger stick BG calibrations, as they are factory calibrated. However, they are still expensive, with or without insurance, because they require a transmitter as well as sensors. Moreover, these sensors could fall off the body or may fail early before the end of the sensor session, and they are difficult to restart after they fail [14-16].

“Tremor” or “trembling” has been reported to be a common sign of hypoglycemic events among patients with diabetes [17,18]. In one study surveying 132 older adults with diabetes, 71% (n=92) reported trembling [19]. Other studies have also shown tremors to be a significant symptom of hypoglycemia whether reported subjectively [20-23] or measured objectively in the lab [24]. No methods are currently available to capture and assess hypoglycemic hand tremors at home. Home monitoring can be a viable tool to provide insight into the tremors and thus help detect hypoglycemia.

Monitoring tremors may provide a cost-effective and nonintrusive method to detect the onset of hypoglycemic events. Accelerometer sensors are validated devices to measure motion and have been used in various applications such as assessing physical activity [25-31], aiding in the management of Parkinson disease [32-34], and gait analysis [35,36]. However, outside of conceptual framework development efforts [37], the only study that attempted to detect hypoglycemia using accelerometer data was our recent work on adolescents with T1DM [38].

Machine learning has shown promise for prognosis in medicine [39]. Supervised machine learning models find patterns across input features to predict the target. With the recent advent of inexpensive wearable physiological sensors, hypoglycemia prediction can be improved. Previous researchers used physiological signals, including photoplethysmography, electrocardiogram (ECG), heart rate (HR), HR variability, galvanic skin response, and skin temperature, to predict hypoglycemic events [6,40-45]. However, the application of machine learning to monitor hypoglycemic events through hand tremors remains a research gap despite the initial promise of extracting physiologic tremor features in adults with T1DM [17,46]. Key barriers to addressing this gap are (1) access to longitudinal tremor data sets in diabetic populations and (2) clinical thresholding of hypoglycemic events based on BG levels.

With these challenges in mind, the objective of this research is to develop machine learning algorithms to detect hypoglycemia through hand tremors using acceleration data from a 1-month home study on adults with T1DM. We expect this research to enable real-time monitoring of hypoglycemia through noninvasive and nonintrusive wearable technologies with a built-in accelerometer sensor. The remainder of this paper describes our methods used to collect data, discusses the data processing steps, presents the results of developed algorithms, and concludes with a discussion of our findings and recommendations for future work.

## Methods

### Data Collection

A home study was designed to collect continuous accelerometer data from participants with T1DM. Accelerometer data were collected using Apple Watch Series 5 (Apple Inc) with a sampling frequency of 64 Hz. We used a mobile app called TremorApp to record, archive, and transfer the accelerometer data. TremorApp is an app our team customized in the lab to run continuously in the background of the watch. It allows participants to make a single tap on the Apple watch whenever they feel they have low blood sugar, and it is logged automatically. In addition, the app is connected to participants’ iPhones, where they can track the number of hypoglycemic events they have reported, as well as their HR and acceleration. The participants then transferred their data from the phone to our cloud folder upon completion of the study.

Participants who had an Apple Watch Series 5 were allowed to use their own watch. We monitored the data for 1 week, and if there were any issues with running the app or data collection, then we mailed them our own Apple Watch Series 5 for the purpose of this study under the agreement that they would return it upon completion.

The inclusion criterion was patients with T1DM who regularly used CGMs. To be consistent, only patients who were using a Dexcom CGM (G5 and G6; Dexcom Inc) were enrolled in the study. Dexcom uses a sensor wire inserted underneath a person’s skin to measure glucose readings in interstitial fluid throughout the day and night, with a sampling frequency of 5 minutes [47].

### Procedures

The participants were instructed to wear the smart watch continuously for 1 month and report the instances of tremors. Every week, participants would upload their accelerometer data file, subjective low blood sugar logs, HR data file, and CGM logs over their phones to a secure server after being trained on how to do so over the internet with the help of a researcher from the team (author KZ). In this study, we only used acceleration data and CGM logs for the classification problem. Self-reported hypoglycemia and heart data were not used in this study.

### Participants

Adults (>18 years old) diagnosed with T1DM who use a CGM device were invited to participate in this study through the university’s campus bulk mail. A total of 45 participants started the study, among whom 7 dropped out due to nonconformance or technical issues with the phone, Apple Watch, or CGM. In addition, 5 patients’ devices did not correctly record accelerometer data. The data collected from 33 patients, including 21 (64%) females and 12 (36.4%) males, aged between 18 and 56 (mean 25.35) years were included in this study. Out of the 33 participants, 3 (9 %) identified as having 2 or more races, and the remaining (n=30, 91%) all identified as White. Additionally, 6 (18%) participants identified as being of Hispanic/Latino heritage. On average, patients wore CGM devices 95.44% (SD 3.27%) of the time per day. Each patient was expected to wear their watch the whole day. However, it was worn 39.93% (SD 29.57%) each day. Therefore, on average, 31.26% (SD 16.52%) of overlapped accelerometer and CGM data equal to 450.14 (SD 237.89) minutes were available per day for each patient. These overlapped data were used in this study. Note that data recorded during sleep were also included and treated the same way as nonsleep data. Additionally, there was no particular period in the day where data were unavailable for all patients. In other words, in every hour of a 24-hour day, there was at least 1 patient with available data.

### Ethics Approval

The study was approved by the institutional review board of Texas A&M University (IRB2019-0261F) and complied with the American Psychological Association Code of Ethics. All participants provided informed consent.

### Data Preprocessing

All data preprocessing was completed using Python version 3.6.9 software (Python Software Foundation). Acceleration components were filtered using a second-order Butterworth low-pass filter (cutoff frequency was set to 30 Hz). The magnitude of the 3D acceleration was calculated as the square root of acceleration components in the x, y, and z directions. To provide sufficient patterns of data for hand tremor detection, accelerometer data were divided into 3-second sliding windows with 50% overlap [48]. Acceleration windows between 150 seconds before and 150 seconds after the CGM sampling were labeled as hypoglycemic or nonhypoglycemic based on their corresponding BG levels. Windows with BG levels less than 70 mg/dL were labeled hypoglycemic, and windows with BG levels between 90 and 140 mg/dL were labeled nonhypoglycemic [49,50]. We also explored sequential classification based on 9 consecutive windows. To facilitate this analysis, only data with 9 consecutive windows were included in the final analysis. After cleaning, labeling, windowing, and consecutive windows consideration, the data set had 89,634.45 minutes of data consisting of 3,585,378 windows with 113,975 hypoglycemic events. One of the challenges of training the algorithms to detect hypoglycemia was the imbalanced data set, with an average of 3.3% hypoglycemic windows per patient. To address this issue, we performed random oversampling (also called “upsampling of the minority class”) by duplicating examples from the hypoglycemic class in the training set [51]. Upsampling was used because it reduces information loss in the quantification process by using the entire data set. In addition, upsampling has proven to be more robust to noise, and it performs better for predictions compared to downsampling [27,52]. Different resampling ratios (1-1, 2-1, 3-1, 3-2, 4-1, 4-2, and 4-3) were evaluated, and the ratio 3 (nonhypoglycemic events) to 1 (hypoglycemic events) was selected based on performance results. Note that oversampling was performed only on the training data, and data were not upsampled in the validation set.

### Feature Extraction

Once signals were preprocessed, a total of 86 features (42 for the time domain and 44 for the frequency domain) were extracted from the windowed acceleration data (x, y, z, and the magnitude) [17,46]. Table 1 provides descriptions and abbreviations of the features employed. Different statistical features were extracted from the time domain, including mean, SD, variance, maximum, minimum, range, number of peaks (NOP), skewness, and kurtosis. The time domain features showed discriminative power for tremor detection [53]. To calculate NOP, we used the mean value of each window as a required threshold of peaks. Additionally, the Pearson correlation coefficients (CORRs) [54] between all combinations of acceleration components (x, y, z) and their magnitudes were computed and used as features. CORR components have been shown to be relevant for tremor detection [55]. In total, 42 features were extracted in the time domain.

The fast Fourier transform was used for the frequency domain analysis. Hypoglycemia is characterized by hand tremors with a frequency range between 4 and 14 Hz [46]. The power spectral density (PSD) of the acceleration windows was calculated using the Welch periodogram [56]. The Welch method computes an estimate of the PSD by dividing the time signal into successive blocks, computing a modified periodogram for each segment, and then averaging the periodograms [56]. Several frequency domain features were extracted from the PSD of all acceleration components x, y, z, and magnitude, including mean, maximum, SD, NOP, average band power (ABP), normalized ABP (NABP), and frequency of maximum PSD (Fmax). The mean of each PSD window was used as a required threshold to calculate NOP. We also calculated mean, maximum, SD, and ABP for the PSD of frequencies between 4 and 14 Hz. We call these features hand tremor frequency range (HTFR) features. In total, 44 features were extracted from the frequency domain of the acceleration data.

**Table 1:** Summary of features included in the machine learning models.

Category and features		Abbreviation
**Time domain**		
	Mean	M
	Standard deviation	SD
	Variance	V
	Maximum	Max
	Minimum	Min
	Range	R
	Number of peaks	NOP
	Skewness	SK
	Kurtosis	KS
	Correlation coefficient	CORR
**Frequency domain**		
	Mean	M
	Maximum	Max
	Standard deviation	SD
	Number of peaks	NOP
	Average band power	ABP
	Normalized average band power	NABP
	Frequency of maximum power spectral density	Fmax
**Frequency domain in 4-14 Hz range (HTFR^a^)**		
	Mean	M
	Maximum	Max
	Standard deviation	SD
	Average band power	ABP

^a^HTFR: hand tremor frequency range.

### Classification Models

Many classification approaches have been used to classify tremors versus normal states, mainly for Parkinson disease or essential tremor disorder [57-64]. However, these approaches have not been applied to tremors caused by hypoglycemia. Tremor studies have used random forest [57-60], support vector machines (SVMs) [60,61,65,66], k-nearest neighbors (KNNs) [58,62,63], and naïve Bayes [58,64]. Among the current approaches, random forest, SVM, and KNN are the most widely used. Comparative analyses in the tremor literature have shown that random forest and KNN models outperform naïve Bayes [58] and perform comparably to SVM [60]. However, because of the diversity in feature extraction methods, ground truths used, and different domains, it is difficult to generalize these findings.

Based on the promise of models used in the tremor literature, we used 3 machine learning models—random forest, SVM, and KNN—to classify hand tremors (hypoglycemic state) from nonhypoglycemic states in patients with hypoglycemia. Randomized searches were performed to tune the models. Random forest is a flexible supervised machine learning algorithm comprising uncorrelated decision trees, which are combined to create more accurate predictions and reduce variance [67]. For random forest, the following hyperparameters were tuned: the number of decision trees in the forest, maximum depth, and the criteria with which to split on each node (Gini or Entropy). Based on the model performance, 100 decision trees with a maximum depth of 5 and the Gini function were used. KNN is a nonparametric algorithm that assumes that similar data points can be found near each other. It seeks to compute the distance (usually through Euclidean distance) between data points and then allocates a category based on the most frequent neighboring data points [68]. A wide range of K neighbors, from 3 to 159, was tested, and finally, K=27 was chosen as it resulted in the best model performance. In addition, Euclidean distance was used to measure the distance between the data points. SVM is typically used for classification problems. In the SVM algorithm, a hyperplane, also called a decision boundary, will be built where the distance between 2 classes of data points is at its maximum. This hyperplane separates the classes of data points on either side of the plane [69]. Different kernel types such as linear, poly, radial basis function (RBF), and sigmoid were tested to map the data set into higher dimensional spaces. The regularization parameter C was also changed from 0.1 to 10, and no significant changes were observed. Finally, an RBF kernel with C=1 was used, as a better performance was observed. Moreover, for all 3 algorithms (ie, random forest, KNN, and SVM), a classification threshold of 0.5 was used.

The 3 machine learning models were trained on the acceleration features. We also used ensemble learning for the hypoglycemia classification. Ensemble methods are techniques that create multiple models and then merge them to improve classification performance [70]. Ensemble methods usually result in more accurate solutions than a single algorithm. We combined random forest, KNN, and SVM for the ensemble learning model. Different approaches exist for the ensemble learning technique, such as majority voting, bagging, boosting, and stacking [71]. We used the majority voting method for the classification task. In this approach, each model makes a prediction (vote) per test instance, and the final output prediction will be the one with more than half of the votes [72].

### Sequential Classification

We performed sequential classification, which is a predictive modeling approach where a consecutive sequence of inputs over time is considered, and the task is to predict the hypoglycemia category for the aggregated sequence as a whole [73]. The inputs were the 3-second windows of acceleration data with 50% overlap. We classified a sequence as hypoglycemia if at least 50% of the 3-second inputs were predicted as such. Otherwise, the sequence was classified as nonhypoglycemia. We tested different sequence times, including 15 seconds, 30 seconds, and 60 seconds containing 9, 19, and 39 windows, respectively. The best performance was obtained for 15-second sequences, and the results reported are based on those sequences.

**Figure 1:**
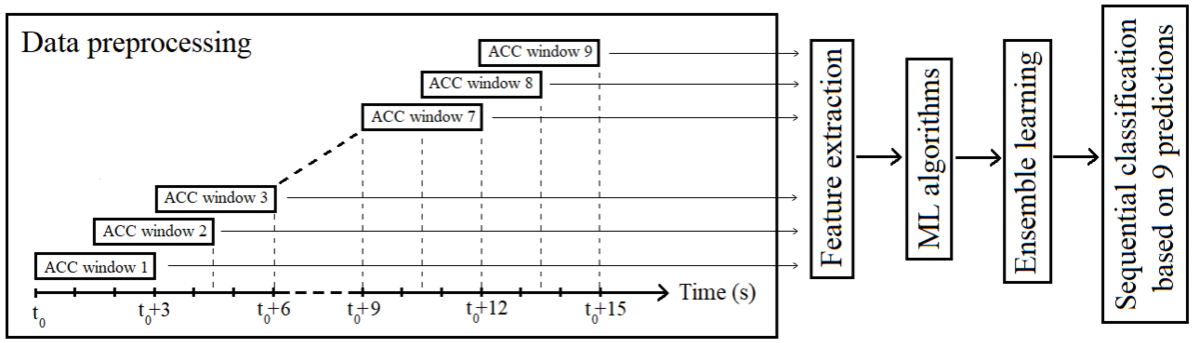
Overview of the analysis approach. ACC: acceleration. ML: machine learning.

All analyses were implemented in Python software (Python Software Foundation). As shown in Figure 1, recordings from 33 patients with hypoglycemia were imported to Python and preprocessed. Time and frequency domain features were extracted from the 3 axes of the acceleration signal and their magnitude. The feature vector was fed to the machine learning models for classification and subsequently for ensemble and sequential models.

### Evaluation

To evaluate the classification models, we used 2 cross-validation (CV) strategies, 10-fold CV and leave-one-subject-out (LOSO) CV [74,75]. The 10-fold CV performed the fitting procedure a total of 10 times, with each fit being performed on a training set consisting of 90% of the data selected at random. The remaining 10% of the data were used as a hold-out set for validation. Note that data from the same participant were not present simultaneously in the training/validation sets. LOSO CV is a special case of CV where the number of folds equals the number of participants in the data set. In this scheme, the learning algorithms are evaluated once for each participant, using all other participants as a training set and the selected participant as a test set. LOSO CV is a robust estimate of model performance, as each participant is given an opportunity to represent the entirety of the test data set [76]. Precision, recall, *F*_1_-score, and accuracy were computed on the validation sets. Precision quantifies the number of positive class predictions that belong to the positive class. Recall quantifies the number of positive class predictions made of all the positive samples in the data set. The *F*_1_-score measures a combination of precision and recall (ie, the harmonic mean of them. Accuracy is the sum of true negatives and true positives over all samples. The following equations define the evaluation criteria used in this study:




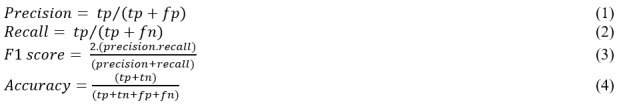




Where *t*, *f*, *p*, and *n* respectively denote true, false, positive, and negative. The hypoglycemia class is considered positive, and the nonhypoglycemia class is negative.

## Results

The mean duration of the hypoglycemic state was 27.31 (SD 25.15) minutes per day for each patient. On average, patients had 1.06 (SD 0.77) hypoglycemic events per day. We used acceleration features in time and frequency domains to classify hypoglycemic versus nonhypoglycemic states through hand tremors. The mean PSD for the frequencies between 4 and 14 Hz 4 for the hypoglycemic windows was 
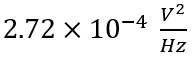
.

However, for the nonhypoglycemic windows, it was 
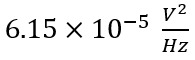
.

**Figure 2:**
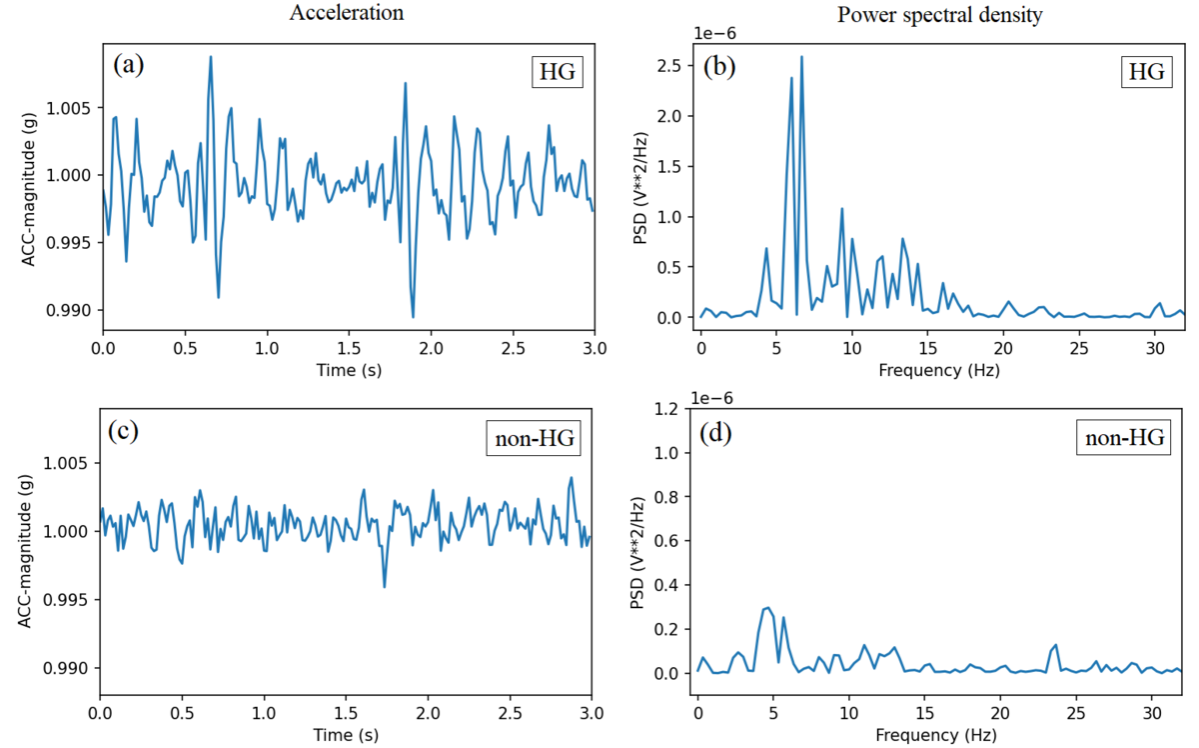
Exemplar acceleration magnitude and the corresponding power spectral density (PSD) for hypoglycemic and nonhypoglycemic states during resting position. ACC: acceleration; HG: hypoglycemic; non-HG: nonhypoglycemic.

**Figure 3:**
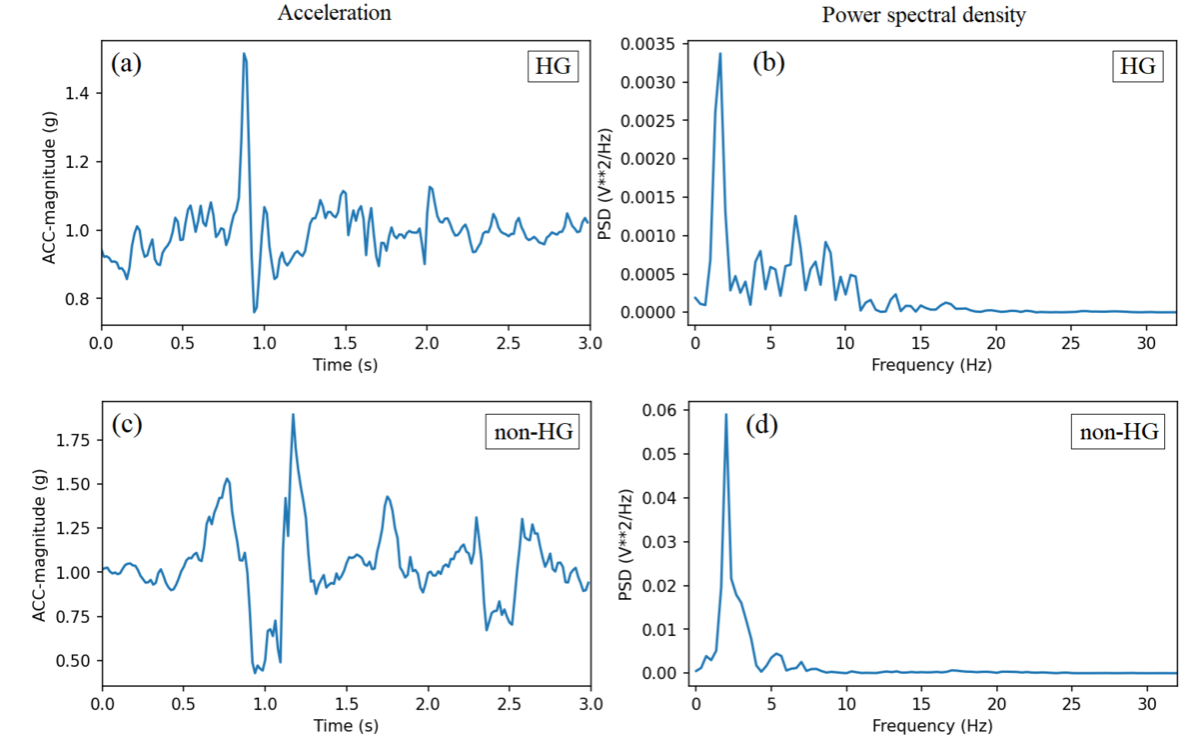
Exemplar acceleration magnitude and the corresponding power spectral density (PSD) for hypoglycemic and nonhypoglycemic states during active position. ACC: acceleration; HG: hypoglycemic; non-HG: nonhypoglycemic.

Figures 2 and 3 show exemplar acceleration magnitude and the corresponding PSD in 3-second windows for hypoglycemic and nonhypoglycemic instances during resting and active positions, respectively. Resting position is when there is no activity; therefore, the acceleration magnitude is close to 1 g. Active position is when the user is moving his/her hand; therefore, the acceleration magnitude is larger than 1 g.

It was observed that in the resting position, the amplitude of the acceleration in the time domain and the amplitude of the frequencies in the tremor range (4-14 Hz) were higher for the hypoglycemic state compared to the nonhypoglycemic state. Additionally, in both resting and active positions, higher variations were observed in the PSD of frequencies between 4 and 14 Hz for the hypoglycemic states than nonhypoglycemic states. The average SD of PSD in frequencies between 4 and 14 Hz for the hypoglycemic windows was 
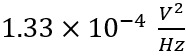
.

However, for the nonhypoglycemic windows, it was 
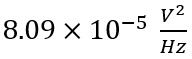
.

Most of the higher amplitude frequencies in hypoglycemic states were in the 4 to 14 Hz range, with some patient-specific variations.

**Figure 4:**
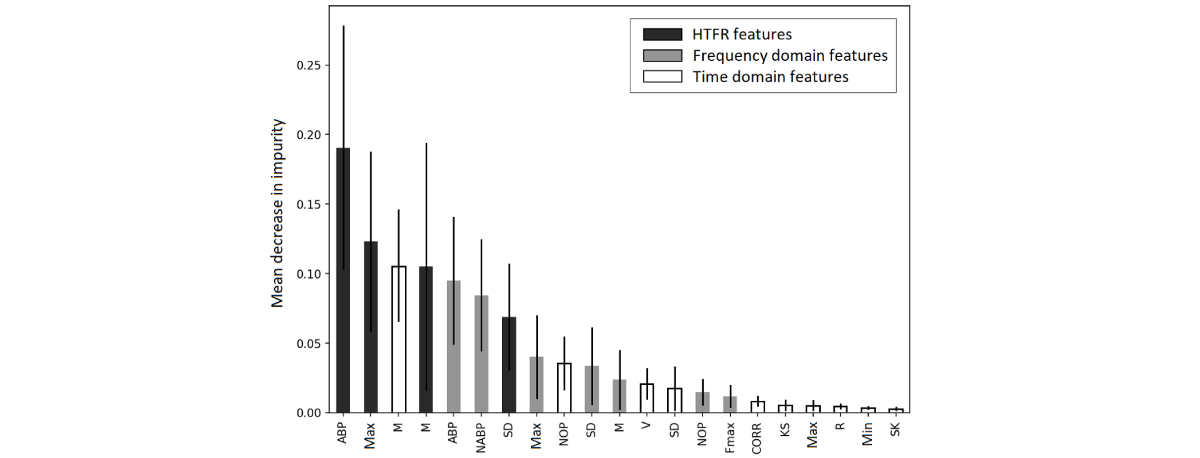
Feature importance using mean decrease in impurity (MDI) from the random forest structure, along with their intertree variability represented by the error bars. ABP: average band power; CORR: correlation between axis; Fmax: frequency of maximum power spectral density; HTFR: hand tremor frequency range; KS: kurtosis; M: mean; Max: maximum; Min: minimum; NABP: normalized average band power; NOP: number of peaks; SK: skewness; R: range; V: velocity.

To better understand which features are more relevant, we computed the mean decrease in impurity (MDI) based on Gini impurity from the random forest algorithm [77]. As shown in Figure 4, the HTFR features and, in particular, the ABP in frequencies between 4 and 14 Hz had the highest importance factors in distinguishing hypoglycemic states. Feature selection was attempted by removing the least relevant features (starting from skewness) based on MDI values shown in Figure 4. Finally, the best model performances were observed when the following time-domain features were excluded from all acceleration dimensions x, y, z, and magnitude: skewness (4 features), minimum (4 features), range (4 features), maximum (4 features), kurtosis (4 features), and CORR (6 features). These features were the least relevant ones based on the MDI values in Figure 4. The results are reported for the feature-optimized classification models based on the remaining 60 features.

**Figure 5:**
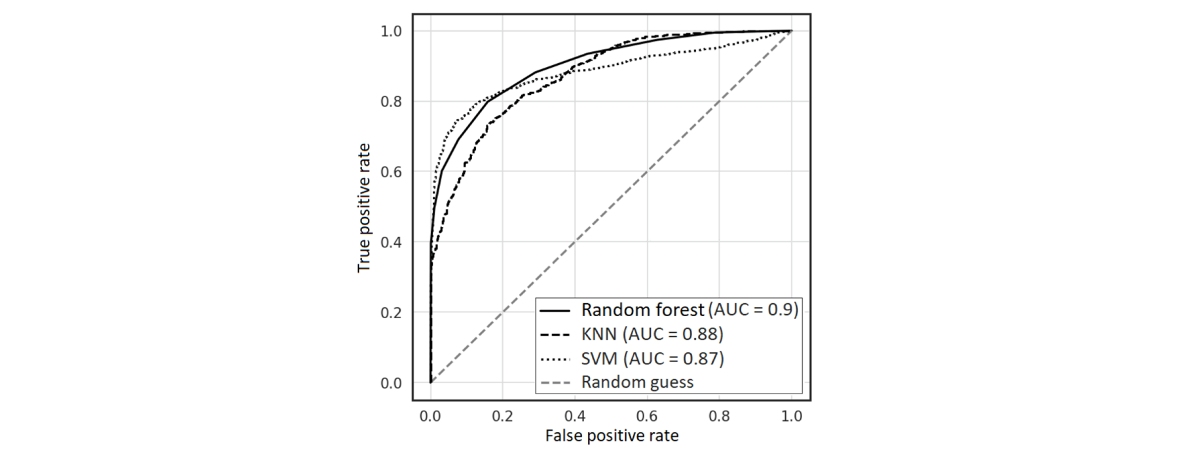
Receiver operating characteristic (ROC) curve and corresponding area under the curve (AUC) values for the 3 algorithms evaluated in this study. KNN: k-nearest neighbor; SVM: support vector machine.

Figure 5 shows the receiver operating characteristic (ROC) curve and the area under the ROC curve (AUROC) for the 3 algorithms using 10-fold CV. The area under the curve (AUC) is a robust measure of binary classification performance since it is not sensitive to class disparities [78]. All algorithms predicted significantly better than random. The random forest model had the highest AUC of 0.9, although the KNN was within 0.02 AUC, and the SVM was within 0.03 AUC. Pairwise comparisons indicated no significant differences between the algorithms.

Table 2 shows the classification performance with all the models based on 10-fold CV and LOSO CV. The random forest model performed better (accuracy of 81.09%, and precision of 82.67%) when evaluated using 10-fold CV, while KNN performed better (accuracy of 79.93% and precision of 82.03%) when evaluated using LOSO CV. The ensemble learning model improved the prediction performance to an accuracy of 81.46% using 10-fold CV and 80.14% for LOSO. The key mechanism for improved performance with ensembles is often the reduction in the variance component of prediction errors made by the models [79]. The ensemble learning model achieved a recall of 78.59%.

**Table 2:** Performance of classification models using 10-fold cross-validation (CV) and leave-one-subject-out (LOSO) CV.

Model	AUROC^a^			Specificity (%)			Precision (%)			Recall (%)			*F*_1_-score (%)			Accuracy (%)		
	LOSO^b^	10-fold	LOSO		10-fold	LOSO		10-fold	LOSO		10-fold	LOSO		10-fold	LOSO		10-fold	
KNN^c^	0.88	0.88	83.15		80.78	82.03		79.92	76.95		76.97	79.41		78.40	79.93		78.83	
SVM^d^	0.87	0.87	81.15		82.93	81.48		79.98	75.94		77.72	78.28		78.83	78.46		80.24	
Random forest	0.88	0.90	80.51		84.48	80.37		82.67	77.45		77.96	78.88		80.24	78.95		81.09	
Ensemble learning	N/A^e^	N/A	81.51		84.55	80.74		81.53	78.82		78.59	79.76		80.03	80.14		81.46	

^a^AUROC: area under the receiver operating characteristic curve.

^b^LOSO: leave one subject out.

^c^KNN: k-nearest neighbor.

^d^SVM: support vector machine.

^e^N/A: not available.

## Discussion

### Principal Results

The primary purpose of this study was to use a wrist-worn accelerometer sensor to detect hand tremors associated with hypoglycemia in patients with T1DM. We used the acceleration and CGM data collected from 33 patients with T1DM. Several machine learning algorithms were employed to develop the detection system. The ensemble learning model achieved the highest accuracy of 81.46%, with 81.5% precision and 78.6% recall for the hypoglycemic class.

Collectively, the results provide support for the use and further development of ensemble techniques (such as random forest), KNN, SVM, or a combination of these models for hypoglycemia hand tremor detection. These results align with previous explorations of tremor detection [50,57,59-61,64,80,81]; however, our findings are focused on hypoglycemia.

### Comparison With Prior Work

The acceleration-based detection system in this study is comparable to the recent research on hypoglycemic detection using other noninvasive sensor-based data such as HR, HR variability, ECG, and temperature. Maritsch et al [82] collected physiological data from patients with T1DM over 1 week using an Empatica E4 smart watch and derived HR and HR variability features to detect hypoglycemic episodes. They achieved a maximum accuracy of 82.7%, with 76.7% sensitivity for hypoglycemic detection using the gradient-boosted decision trees algorithm and 10-fold CV. Elvebakk et al [83] used multiple sensors to collect sudomotor activity data at 3 skin sites, ECG-derived HR, HR-corrected QT interval, near-infrared, and bioimpedance spectroscopy data from 20 patients. They found that hypoglycemia could be identified with a maximum *F*_1_-score accuracy of 88%. Marling et al [40] used HR, galvanic skin response, and skin and air temperatures collected over 2 months to detect hypoglycemia in patients with T1DM who were middle-aged. They showed that an SVM model with a linear kernel could differentiate hypoglycemic from nonhypoglycemic states. Porumb et al [84] used a personalized medicine approach and deep learning models, convolutional neural network, and recurrent neural network, to automatically detect nocturnal hypoglycemia using a few heartbeats of raw ECG signals recorded with wearable sensors. They achieved a maximum accuracy of 85.7% and sensitivity of 84.7% for hypoglycemia detection using their proposed convolutional + recurrent system. The presented model in our study achieved a maximum accuracy of 81.46%, with 78.82% recall for hypoglycemic detection solely relying on a wrist-worn accelerometer sensor.

### Strengths, Limitations, and Future Work

The method documented in this paper represents our initial computational work for detecting hand tremors associated with hypoglycemia using acceleration data in a naturalistic setting. To our knowledge, this is the first paper documenting the application of machine learning for the detection of the onsets of hypoglycemia using hand tremors. We used a longitudinal data set collected within 1 month, comprising 21 females (64%) and 12 males (36%), with an average of 24.04 and 26.26 minutes hypoglycemic per day, respectively. The obtained results suggest that wrist-worn accelerometers may provide the necessary sensory information to detect the presence of hand tremors associated with hypoglycemia. Given the increased availability, affordability, discreetness, accuracy, and nonintrusiveness of smart watch–based accelerometer sensors, these results show promise as an alternative to CGM for the early detection of hypoglycemic events, and they may have life-saving implications.

However, this study is not without limitations. First, the analysis presented here is based on a limited sample. In addition to the 5 patients (13%) whose devices did not adequately record their accelerometer data, 3 (9%) patients did not have any low blood sugar readings recorded on their CGM. This might be due to some CGM users setting higher thresholds for hypoglycemic alerts (eg, 75-80 mg/dL), perceiving hypoglycemic events early, or better managing hypoglycemic events. In addition, participant age could also be an important limitation since most of the participants in this study were college students with an average age of 24.56 (SD 9.67) years.

HTFR features were extracted from the PSD between 4 and 14 Hz frequencies to distinguish hypoglycemic states from nonhypoglycemic states. Although HTFR features helped improve the classification performance, there were several windows labeled hypoglycemic without showing noticeable power density in the 4 to 14 Hz frequencies and several windows labeled nonhypoglycemic with high power density in the 4 to 14 Hz frequencies. Different reasons can cause these to happen during accelerometer or CGM readings, such as motion artifacts or nonhypoglycemic tremors. This study collected data during activities of daily living. Motion artifacts are unavoidable when an acceleration sensor is used in dynamic conditions. Sensor measurements are usually contaminated by motion artifacts due to hand movement, wearable tightness level [85], physiological tremors [86], and so on. Noise will become more critical with the smart watch’s tightness level. When worn too loosely, the device will frequently slide along the wrist, thus negatively impacting sensor accuracy. The effect of the tightness in terms of signal quality will be exacerbated during high-intensity activities.

People who experience hypoglycemic events are likely to experience repeated episodes of hypoglycemia. Over time, repeated episodes of hypoglycemia can cause hypoglycemia unawareness. The brain and body no longer produce symptoms that warn of low blood sugar, such as tremors or irregular heartbeat [87,88]. The approach proposed in this paper is not capable of capturing such events. Another limitation was that the hypoglycemia threshold is personal, and it can change based on the physical activity level [89]. In this study, the patient definition (personalized threshold) of hypoglycemia was not available. Therefore, for all patients, we used an average value of 70 mg/dL, which is commonly cited as a threshold of hypoglycemia for many people [7,90-92] and is the clinically prescribed threshold for hypoglycemia [93]. Future work should set a personalized threshold of hypoglycemia for different patients to capture this event accurately.

In this study, we do not distinguish between the different causes of low glucose values and hand tremors. For example, high-intensity physical activities may cause blood sugar to drop below this threshold in some instances [94]. Future work should explore activity-aware methods to remove such instances from the hypoglycemia class to improve the performance of learning algorithms. In addition, hand tremors may be induced by either toxins (such as excess of certain heavy metals in the body) or medications (such as antidepressants) [95], or they may be related to essential tremors [96]. Therefore, future studies should account for these potential confounding factors in recruitment and analysis efforts. Future work may also analyze additional measures, such as HR variability [97,98], to differentiate hypoglycemic events from nonhypoglycemic events and improve the performance of learning algorithms.

Finally, the objective of this research was not to evaluate an intervention. As a result, participants were not instructed to undertake any particular action to manage hypoglycemia (such as eating or drinking certain foods) beyond their normal habits. However, the findings documented in this paper can inform the design of nonintrusive accelerometer-based hypoglycemia detection and monitoring tools and systems.

### Conclusion

Hypoglycemia is a prevalent disease that affects millions of people worldwide. While tools and technologies exist to help patients with hypoglycemia monitor their BG, they are either invasive, requiring finger pricking, or intrusive and expensive. The proposed work utilized a combination of nonintrusive and noninvasive sensing and machine learning methods to develop detection algorithms for hypoglycemic events via hand tremors. This paper documents the potential of linear accelerator data to provide significant utility for classification models that detect hypoglycemic hand tremors and distinguish between hypoglycemic and nonhypoglycemic states. Our results, while preliminary, suggest that wearable monitoring technology for the continuous detection and remote monitoring of hypoglycemic events through hand tremors is an achievable goal in the near future.

## Abbreviations

ABP: average band power
AUC: area under the curve
AUROC: area under the receiver operating characteristic curve
BG: blood glucose
CGM: continuous glucose monitor
CORR: correlation coefficient
CV: cross-validation
ECG: electrocardiogram
Fmax: frequency of maximum power spectral density
HR: heart rate
HTFR: hand tremor frequency range
KNN: k-nearest neighbor
LOSO: leave one subject out
MDI: mean decrease in impurity
NABP: normalized average band power
NOP: number of peaks
PSD: power spectral density
RBF: radial basis function
ROC: receiver operating characteristic
SVM: support vector machine
T1DM: type 1 diabetes mellitus
